# Task Shifting and Task Sharing to Strengthen the Surgical Workforce in Sub-Saharan Africa: A Systematic Review of the Existing Literature

**DOI:** 10.1007/s00268-023-07228-6

**Published:** 2023-11-11

**Authors:** Alex J. van Duinen, Adam Gyedu

**Affiliations:** 1 Institute of Nursing and Public Health, Norwegian University of Science and Technology, Trondheim, Norway; 2 Department of Surgery, ELWA Hospital, Monrovia, Liberia; 3 Department of Surgery, School of Medicine and Dentistry, Kwame Nkrumah University of Science and Technology, Kumasi, Ghana; 4 University Hospital, Kwame Nkrumah University of Science and Technology, Kumasi, Ghana

## Introduction

Lack of human resources remains a major challenge in adequate provision of accessible and safe surgical services when needed. Lack of access to surgery results in avoidable morbidity and mortality, and poor and marginalized populations are affected the most. In areas where there is a lack of surgical specialists, task sharing and task shifting has been suggested as relevant strategy to improve access to surgery [1]. Task shifting can either be done from specialist to non-specialist medical doctors (first degree) and from medical doctors to associate clinicians (second degree). In certain areas, such as obstetrics and ophthalmology, task sharing has been documented as a safe strategy [2].

Ryan et al. provide a rigorous overview of the existing literature regarding task shifting and task sharing in the domain of general surgery in sub–Saharan Africa [3]. Most of the articles that were assessed for clinical outcomes demonstrated non-inferiority of procedures performed by non-specialist clinicians (1st and 2nd degree combined), although there was heterogeneity with respect to non-specialist clinician training, surgical cases, and variables studied. The authors conclude that task sharing, and task shifting is a potentially cost-effective solution for the lack of surgeons. Does this open the door for a wider implementation of task shifting and sharing?

If the surgical community wants to take universal health coverage seriously, the role of task shifting and sharing needs to be considered. The number of general doctors and surgeons in sub-Saharan African countries is improving. However, many of these countries currently train too few specialist surgeons to reach the threshold of 20 per 100,000 population in the near- or medium-term. Thus, increasing the capacity of surgeons alone will not be sufficient to provide adequate access in the interim and task shifting/sharing should be considered.

Sierra Leone started in 2011 with a task sharing program where non-specialist medical doctors and associate clinicians are trained in basic life-saving obstetrics and surgery. The training program has contributed to strengthening the human resources in surgery, especially in the district hospitals. Even though, the graduates of the program are contributing substantially to the national surgical volume, there is still a big unmet surgical need [4]. One of the most important elements of this training is the emphasis on teamwork. Tasks are done better and more effectively when shared in a team compared to done by individuals. In a surgical team, different cadres have different capacities, roles, and responsibilities. When this is accepted and utilized well, the team can provide better patient care leading to improved surgical outcomes.

However, regulating the activities of non-specialist medical doctors and associate clinicians remains a challenge, especially in rural facilities where continuous oversight may be limited. In addition, the scope of practice is not always clearly defined. To protect patient safety while upscaling task sharing implementation, a more coordinated effort for standardization is needed. For obstetrics, WHO has provided clear guidance on the implementation of task shifting and a similar process for general surgery would be beneficial [5].

Task shifting and sharing are opportunities to improve access to surgical services. The surgical community should move from the question of its safety towards how to optimally implement this strategy.
